# A Pragmatic Approach for Developing Landbase Cumulative Effects Assessments with Aggregated Impacts Crossing Multiple Ecological Values

**DOI:** 10.1007/s00267-022-01632-9

**Published:** 2022-03-28

**Authors:** Glenn D. Sutherland, Jason Smith, F. Louise Waterhouse, Sari C. Saunders, Kathy Paige

**Affiliations:** 1 Wildlife Infometrics Inc., #3-220 Mackenzie Blvd., Mackenzie, BC V0J 2C0 Canada; 2 Ecora Engineering and Resource Group, #200-2045 Enterprise Way, Kelowna, BC V1Y 9T5 Canada; 3 B.C. Ministry of Forests, Lands, Natural Resource Operations and Rural Development, Coast Area, West Coast Region, #103-2100 Labieux Rd., Nanaimo, BC V9T 6E9 Canada; 4 B.C. Ministry of Environment and Climate Change Strategy, Ecosystems Branch, 525 Superior St., Victoria, BC V8V 0C5 Canada

**Keywords:** Cumulative effects assessment, Valued components, Environmental response factors, Indicators, Benchmark conditions, Aggregated impacts

## Abstract

In strategic cumulative effects assessments, significant methodological challenges exist for classifying and aggregating impacts when using multiple indicators to determine relative risks upon ecological values from anthropogenic developments. We present a strategic spatial modeling case study CEA (2012–2112) in a 909,000 ha forested landscape of Southwestern British Columbia. We explore decisions needed to calculate and aggregate modeled indicators of cumulative anthropogenic footprints on landscape conditions by examining the choice of quantitative methods. We compare how aggregated impact conclusions may differ for seven indicators grouped in two ways to represent three ecological values (Forest Ecosystems, Riparian Ecosystems and Species at Risk): four expert-defined policy-driven valued components (VCs) or three analytically derived environmental resource factors (ERFs). By explicitly demonstrating methodological choices at each step of impact estimation and aggregation, we outline a practical systematic approach to customize strategic CEAs of this type and retain transparency for interpreting impacts among values. Aggregated impacts for VCs appeared dominated by those estimated from “condition” indicators describing the degree of expected deviations in indicator states from desired conditions; aggregated impacts of ERFs were dominated by “pressure” indicators linked to underlying causal processes assumed important for describing changes in future ecological conditions. High spatial congruence occurred between impact statements for some VCs compared to ERFs representing the same ecological value; poor congruence between others likely occurred because they represented different ecological processes. Aggregated impact classifications may usefully signal impact severity and risk but are dependent on indicator grouping, hence choices for aggregation are integral to the assessment process.

## Introduction

Cumulative effects assessment (CEA) describes and quantifies the impacts of accumulated and sometimes interacting effects from natural and anthropogenic stressors upon responses of ecological systems over time and space (Canter and Ross 2010; Noble et al. 2011; Duinker et al. 2013; Dubé et al. 2013; Olagunju and Gunn 2015). Comprehensive methods for assessing risks of future development trajectories upon the wide spectrum of terrestrial and aquatic values involved in strategic landscape planning continues to pose significant methodological challenges (Opon and Henry 2020; Mahon and Pelech 2021; Venier et al. 2021). CEAs typically identify potential disturbances (stressors) caused by a proposed project, identify responses in the environment caused by those stressors, and assess the interactions between stressors and environmental responses to stress (Dubé 2003; Venier et al. 2021). Here, we are specifically interested in how these steps can be undertaken when assessing impacts on ecological values resulting from potential cumulative anthropogenic footprints projected forward in time (Sutherland et al. 2016; Venier et al. 2021).

Identifying comprehensive and systematic methods for linking stressors with indicators’ responses to them, and then estimating impacts on components of selected ecological values remains elusive (Bragagnolo and Geneletti 2012; Squires and Dubé 2013; Opon and Henry 2020). Components of ecological values are variously referred to as valued ecosystem components (Beanlands and Duinker 1984); valued components (VCs: e.g., Sutherland et al. 2016), or environmental response factors (ERFs: Venier et al. 2021). Difficulties in calculating impacts include a frequently encountered lack of data on relationships between stressors and indicators, choosing appropriate reference conditions to use when inferring the ecological meaning of deviations from them (Recatalá and Sacristán 2014; Duinker et al. 2013) and finding composite indicator groupings that more clearly represent underlying ecological processes (Recatalá and Sacristán 2014; Sutherland et al. 2016; Venier et al. 2021). The desired goal is to estimate impacts for individual or multiple indicators in order to evaluate the likelihood of adverse ecological effects (i.e., ecological risks) occurring as a result of stressors acting upon the biological functioning and long-term sustainability of the desired landscape or ecosystem.

An ongoing challenge for CEAs is determining the extent to which aggregating evidence obtained from multiple indicators can in fact lead to transparent assessments of present and future risks to environmental and other values in the context of long-term policy goals (Sutherland et al. 2016; Sinclair et al. 2017). More specifically, can analyses of (aggregated) responses in indicators be structured to transparently assess impacts among diverse ecological values (Mahon and Pelech 2021)? Other challenges include uncertainties about the types of relationships and strengths of interactions between indicators when assessing effects on VCs or ERFs (Sutherland et al. 2016; Opon and Henry 2020). Lack of sufficient background data (Wu et al. 2015), identification of meaningful spatiotemporal scales (Gan et al. 2017; Venier et al. 2021), and potential masking effects occurring during aggregation (Duinker and Greig 2006; Therivel and Ross 2007; Bragagnolo and Geneletti 2012) in combination with the inherent complexity of interconnected relationships between ecological processes (Hegmann 2019; Opon and Henry 2020) are all problematic for a transparent modeling approach such as ones used in assessing impacts of resource development patterns on landscapes.

In this paper, we develop a pragmatic and adaptable methodology for analyzing, estimating and evaluating the potential cumulative effects of forestry and related resource development footprints upon VCs or ERFs as measured by changes in multiple indicator metrics. We examine and evaluate how impacts could be aggregated, and the dependency of the impact conclusion on the indicators selected to represent these values using VCs or ERFs. We review key decision points and demonstrate our selection of the most parsimonious options for undertaking: (1) impact calculation (e.g., reference conditions, benchmarks), and (2) aggregation of impacts (e.g., effects of aggregation criteria, calculation approach) using strategic landscape modeling tools. Our primary goal is to convey a systematic approach that selects practical options from among those available for aggregating multiple indicators into combined impact statements for assessing ecological risks. As well, we explore how options for aggregation are affected by the characteristics of the indicators and their grouping (e.g., VC or ERF).

## Methodology

### Overview of Research Case Study

#### Study area

The research case study was based on the landscape conditions and scenarios of potential future development trajectories for a 909,000-ha area in southwestern British Columbia, Canada (Sutherland et al. 2016). This area is topographically and ecologically diverse, and has historically supported a range of economically valuable resource-based activities (i.e., forestry, small-scale hydrological energy production, agriculture, fishing, recreation). The area is also important for the conservation of old-growth forests and contains important habitats and populations of several species at risk under Canada’s Species at Risk Act. Because of the steep terrain, infrastructure footprints tend to be concentrated in valleys and estuaries, heightening the potential for cumulative impacts upon multiple ecological values.

#### Spatiotemporal projection models and scenarios

We forecasted potential future landscape conditions using a set of raster-based spatiotemporal simulation models implemented in SELES^1^ (Fall and Fall 2001; Sutherland et al. 2007) at a 0.25 ha spatial resolution, except for models involving streams, which used a 0.0625 ha resolution. These models make annual projections from an initial year (*t*_0_; in this case 2012) to 100 years into the future (*t*_100_; 2112) simulating vegetative changes and footprints (extents) of both natural and anthropogenic disturbances at each location (raster cell). Six linked spatiotemporal simulation models were used to project an annual time series (*t*_0_,…,*t*_100_) of indicator metrics at two spatial scales (landscape or watershed) and are as follows:

1. tree growth—incrementing ages and heights for the identified leading and co-dominant tree species defining the forest stand at each location;
2. forest harvesting and silviculture—modifying stand descriptors and landcover type based on a spatial harvest and silvicultural prescription schedule;
3. natural disturbances—probabilistic simulation of landscape-level wildfire dynamics and effects on stands;
4. run-of-river (RoR) developments—probabilistic implementation and decommissioning of small run-of river power projects and their associated infrastructures (penstock, powerhouse, roads and transmission lines);
5. access connectivity—road and transmission line submodels that connect and extend existing road and transmission line corridors as required by the different anthropogenic developments. These are implemented at each time step according to rules consistent with these types of infrastructures; and
6. indicator generation—summarizes the projected indicator values at the different scales of interest for cumulative effects analyses.

A more complete description of the model inputs, parameter values, and descriptions of these models are given in Supplementary Information S1.

Two scenarios for calculating effects on indicators and aggregating impacts across multiple values (see Supplementary Information S2) were each run once for the case study, as follows:

1. Long-term equilibrium (LTE)—recreates assumed “baseline reference conditions” using projections of historical patterns of stand-replacing wildfires with no resource development or fire suppression. Disturbance footprints from projected wildfires or current harvested stands are replaced over time (1100 years) with old forest, unless new stand-replacing wildfires are initiated.
2. Full development—projections of forest harvesting and RoR projects for our indicator projections and impact calculations. Harvest levels are projected as under the May 12, 2011, Timber Supply Review Allowable Annual Cut determination (BC Ministry of Forests and Range 2010). RoRs are projected for years 1–20 using water license data; for years 21 onwards using BC Hydro RoR potential mapping, both with a parameter specifying incremental annual probabilities of initiating a new development site.

### Indicator Selection

Seven watershed-scale indicators representing the ecological values of forest ecosystems, riparian ecosystems, and species at risk were selected. Indicators for analyses were categorized as “condition” indicators (e.g., Old forest area) describing the degree of an expected change in the condition (state) at a point in time, or “pressure” indicators (e.g., road densities) that describe the estimated level of development pressure affecting condition, and which may be causally important in defining future conditions. Following methods in Sutherland et al. (2016), these seven indicators were grouped into: (1) four pre-selected policy-driven VCs: Old forest condition, Riparian condition, Stream condition, and Spotted Owl (*Strix caurina occidentali*s) habitat condition, each addressing important management objectives and societal values (Table 1), or (2) regrouped using PCA analysis as ERFs specifically Road disturbance, Old forest retention and recruitment, and Spotted Owl habitat state.

**Table 1:** Seven indicators grouped to represent four policy-driven valued components (VCs) or three analytically derived ecological response factors (ERFs)^a^ evaluated at the watershed scale

Indicator	Indicator type	Reference condition	Indicator interpretation		Ecological value	VC	ERF
Old forest area (%)	C	LTE^b^	Percent of total area that is forested and >250 years old^c^	Forest ecosystem		Old forest condition	Old forest retention and recruitment
Density of active roads (km/km^2^)	P	Current	Density of all roads (current or projected)			Old forest condition	Road disturbance
Density of transmission lines (km/km^2^)	P	Current	Density of all transmission lines (current or projected)			Old forest condition	Spotted Owl habitat state
Road density on coupled steep slopes (km/km^2^)	P	Current	Density of roads on steep slopes (>60%) that extend to within 100 m of a stream	Riparian ecosystem		Stream condition	Road disturbance
Hydrological recovery (ha/ha)	C	Current	An indicator of peak flow, based on the height of the dominant trees in a stand, where the taller the trees, the greater the hydrological recovery			Stream condition	Old forest retention and recruitment
Density of stream crossings by roads (#/km^2^)	P	Current	An indicator of expected sediment load			Riparian condition	Road disturbance
Spotted Owl nesting habitat area (%)	C	LTE	Nesting habitat based on stand age, tree height, elevation, and biogeoclimatic ecosystem type (see Sutherland et al. 2007)	Species at risk		Spotted Owl^d^ habitat	Spotted Owl habitat state

Indicator types are C = condition; P = pressure (see text for definitions)

^a^More detailed descriptions of indicators are available in Sutherland et al. (2016), in which the term “ecological function” was used instead of “ecological response factor (ERF)” here. The terms are equivalent in meaning

^b^Historically undeveloped condition, modeled using the long-term equilibrium (LTE) scenario (see text for details)

^c^We used age criteria (stands >250 years old) to define “Old forest” in our study area using mapping from Vegetation Resource Inventory (BC Ministry of Forests and Ministry of Environment 1995)

^d^Spotted Owl is the Northern Spotted Owl (*Strix caurina occidentalis*), an imperiled species at risk occurring in this study area. Potential Spotted Owl nesting habitat is usually >250 years old but in some drier ecosystems it can be younger (e.g., minimum age 110 years; Sutherland et al. 2007)

### Estimation of Impacts

**Fig. 1:**
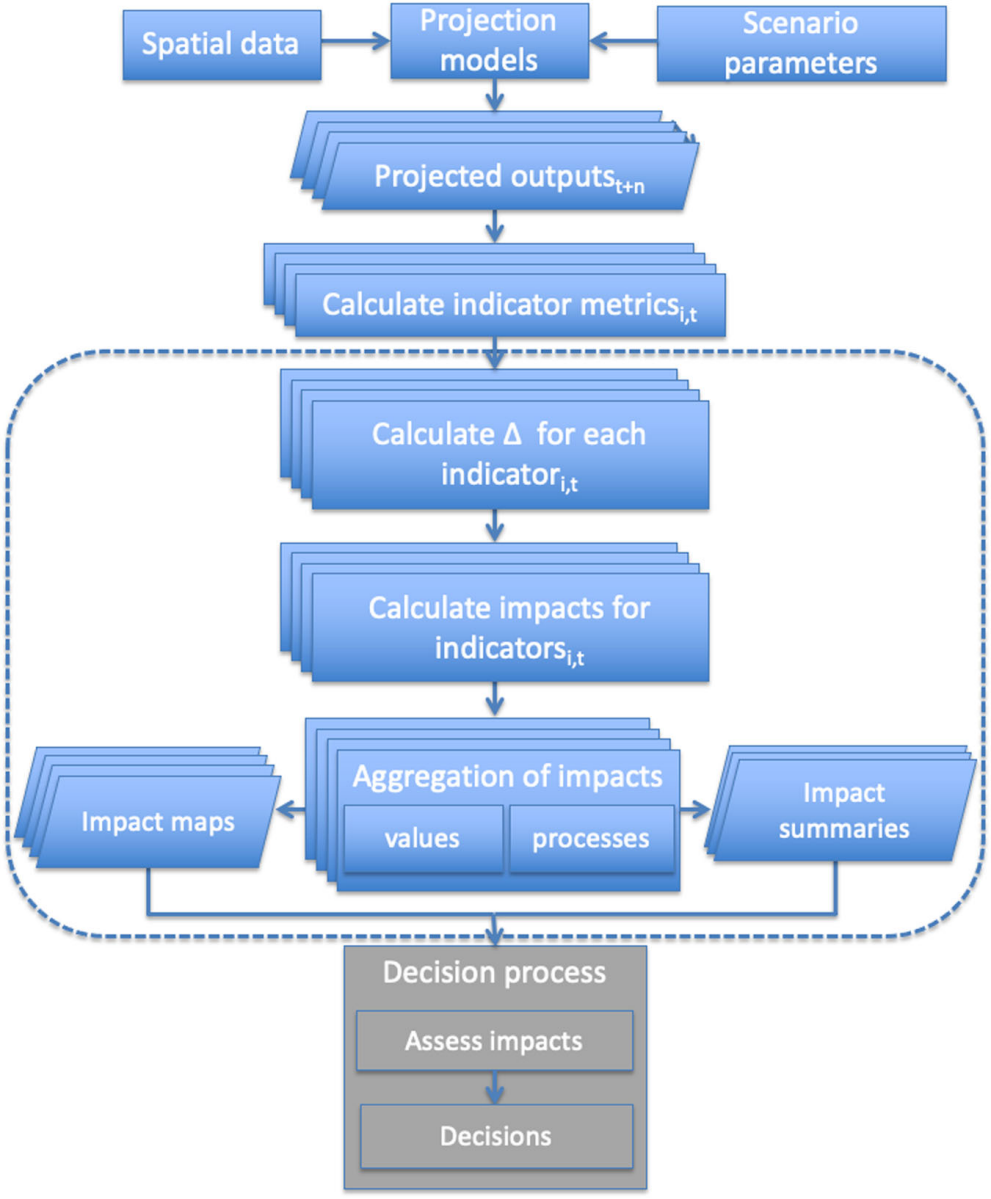
Conceptual diagram of an overall framework for projecting, aggregating, and interpreting impacts for CEAs involving multiple indicators and ecological values using strategic modeling of projected anthropogenic disturbances in landscapes. The dotted rectangle encloses the components of the methodology that is the focus of this paper. This sequence relates to calculating projected changes in indicators, evaluation of impacts, and then aggregating those impacts into an overall impact class at the VC or ERF level. See text for definitions.

Figure 1 describes the sequence of steps used for the landscape modeling approach. Metrics for the time series of seven selected indicators were projected at the raster scale, and results were calculated at the watershed scale. The study area is made up of 243 watersheds of which 142 are entirely contained within the study area boundary. Watershed is the finest spatial scale (extent) for estimated impacts; landscape scale refers to the entire study area.

#### Step 1—calculating metrics of change for each indicator

Cumulative changes (Δ) in the values of each indicator are calculated from the time series of metrics generated by modeled scenarios. To quantify the cumulative impacts of anthropogenic activities within a watershed or landscape, a reference condition must be established against which to measure the deviation of indicator values under the chosen scenario of development (Ball et al. 2013). Ideally, the chosen reference condition will reflect a desirable state or condition of the landscape given the objectives of management policy and of the analysis of cumulative effects.

Use of the current condition of the landscape is one approach for defining a reference condition, but often the landscape objective is to minimize further deviation from “natural conditions” or “naturalness” (Stoddard et al. 2006). Historically undeveloped conditions or “naturalness” excludes most anthropogenic disturbances but includes natural disturbance events (e.g., wildfires, windthrow events, geomorphological changes). Thus, reference conditions for each indicator would ideally be given by the state of the landscape prior to industrial activity (e.g., pre-European state for our study area). However, because historic anthropogenically undisturbed conditions are usually unknown, a second option may be to choose a “best available” or “best attainable” condition (Reynoldson et al. 1997; Stoddard et al. 2006, respectively). This can be created by extrapolating empirical data, if available and representative, from similar sites that are relatively undisturbed by human activities.

A third option, specific to strategic modeling CEA, is to model the dynamic relationships between multiple indicators and landscape attributes to produce an estimate of indicator values under proposed reference conditions. This predictive model-based approach assumes that there is sufficient confidence in the parameters generating the reference conditions, as well as in the representation of cause–effect relationships linking landscape conditions to indicators, to generate meaningful and comparative indicator values. Strategic landscape modeling generally lends itself well to measuring changes in the condition of ecosystems and habitats that are described by terrestrial features linked to projectable attributes, such as trees and roads. Prediction of effects on indicators of air or aquatic values (e.g., water quality) may be more limited by lack of adequate models and/or needed parameters.

For our study, we selected two potential reference conditions for the landscape:

1. Current conditions—deviations from the current condition of the landscape (2012) provides a practical reference condition because it can usually be measured. However, where significant ecological impacts have already occurred, such as in heavily developed areas, impacts may be underestimated, at least at some scales.
2. Historically undeveloped conditions—deviations from undeveloped conditions recreated using the LTE landscape scenario is consistent with both ecological theory and ecosystem-based management principles (Price et al. 2009) and appropriate to our selected VCs or ERFs.

The reference condition we selected to represent effects for each indicator varied based on indicator type (Table 1).

Given these two reference conditions, we considered three options for calculating changes in projected indicator values over time:

1. Use the raw values of the projected indicators, and compare these directly to the reference values (Joseph et al. 2017), for example (*X*_*itn*_− *R*_*ito*_) where *R* is the chosen reference value for indicator *ji* at time t_0_. This approach may be applicable for those types of indicators that are independent in describing the VC, directly measurable on site, and where regulatory guidelines specify empirical target values (e.g., water chemistry measurements) or policy-driven management targets.
2. Standardize the measurement scale for each indicator, such that their projected values are represented as a proportion between a minimum and a maximum observable value. This approach enables aggregation, but requires that the minimum and maximum values for each indicator are known and the biological relevance of these values can be assessed.
3. Calculate the % difference of the projected value of each indicator relative to its reference value(s). This approach is the most general and requires the fewest assumptions. Therefore, we suggest this approach is most often appropriate for using with projected estimates when aggregating multiple indicators using landscape modeling.

Here, we applied the third option (change in projected value from reference value for each indicator) as there were few specified regulatory target values established in policy, and no maxima or minima available for our suite of indicators. Using the projected time series of values for our subset of indicators representing VCs or ERFs (Table 1) at each raster cell, we calculated cumulative changes (Δ) in indicator metrics (*X*_*i*_) from the identified reference condition.

A common challenge for modeling impacts with anthropogenic footprints is dealing with small or zero indicator values, because the level of impact between watersheds may not be comparable if areal extent of watersheds varies and amount of disturbance varies. This may become particularly problematic if impacts of small disturbances are masked. Our solution to this challenge was to remain precautionary and assume that the first problem—identifying cumulative changes in indicators away from an undisturbed, negligible reference condition—is very important to identify. Therefore, we flagged the watersheds in our modeled landscape in which this situation occurred, and classified their estimated impact as being “high” in our calculations of area-weighted impacts in Step 2 below.

#### Step 2—calculating effects (impacts) for each indicator

Determining thresholds for estimation of effects and assessing their significance is currently a major scientific challenge in CEA (Johnson 2013; Johnson and Ray 2021; Venier et al. 2021) given the frequent paucity of research data and unknown dependencies among factors. Magnitudes of change are often defined in terms of discrete classes or states (e.g., impacts are likely to be “moderate”) that can be more easily interpretable thus informative for decision makers (e.g., a 55% change in indicator *X* relative to a “no impact” value). This approach requires that changes in indicator values from reference conditions can be classified to represent different significance classes of impact (e.g., Probst and Stelzenmüller 2015) with benchmarks being the indicator value at the transition point to a new impact class. These benchmarks quantify the tolerance to change in the underlying indicators of a VC or ERF that identify if and when deleterious or hazardous consequences may become likely. Under a discrete state-based approach, the interpretation of the relative magnitude of different effect classes is dependent on the often unknown shape of the indicator’s response to disturbance (e.g., linear or curvilinear); however, some information on the direction and shape of the relationship between indicators and impacts is needed and expert opinion is often required. Effect classes can be used for identifying positive impacts as well as negative ones. Benchmark values to define impact classes are usually characterized three ways: (1) empirical—empirical data are available to define the type of benchmark (e.g., linear features: Seiler and Folkeson [2006]) and potentially also the shape of the effect-response curve; (2) general—a set of benchmarks consistent with ecological threshold literature reviews for the indicators (see Price et al. 2007); and (3) precautionary—a narrower set of deviation benchmarks consistent with the risks associated with some types of anthropogenic disturbances, and/or spatial locations already identified as being sensitive to additional disturbances.

In our case study, we applied a discrete state-based approach to characterize the significance of effects of changes (Δs) in indicator values through time as an effect class or state. We considered two threshold values to define classes: (1) one below which impacts would be considered “low” (impact state = 1); and (2) one above which impacts would be considered “high” (impact state = 3). Between these values, impacts are considered moderate (impact state = 2). Because sufficient empirical data were lacking for our set of indicators, we chose a “general” set of benchmarks for our analyses (i.e., <30% of reference as “high”; 30–70% as moderate, and >70% as “low”; Price et al. 2007; Price and Daust 2009). We used these to calculate indicator impact classes for each watershed as this scale is of primary interest to decision makers. We determined impact classes for each indicator based on the changes in indicator values at each time period (i.e., ∆*X*_*jtn*_) in relation to a chosen benchmark value for each unit (e.g., watershed) in the study area using an area-weighted average of the ∆*X*_*jtn*_ based on the rasters in each watershed.

#### Step 3—aggregating effects into impact classes by VC or ERF

Once impacts are classed for each indicator, the classes may be combined spatially to assess an aggregated impact state for a grouping of indicators representing one or more VCs or ERFs. However, care is needed when considering if and how this type of impact aggregation (or “roll-up”) is undertaken to ensure that the results are informative and useful at the strategic or regional scale. The approach for grouping indicators depends on the characteristics of the indicators and on the selection of VCs or ERFs (Sutherland et al. 2016).

There are two assumptions about the functional relationships among indicators that must be specified:

1. Relative weight applied to each indicator for aggregation of impacts. Choice of weighting method and assigned weights can have a significant impact on the final impact classification (Dobbie and Dail 2013; Becker et al. 2017; Gan et al. 2017; Opon and Henry 2020). In general, there are two different weighting methods:
  - Equal weight: equal weighting for each VC or ERF indicator, assuming each signals an independent and equally important potential change. This was the approach we followed in our case study.
  - Variable weight: three alternative methods for applying variable weights to indicators of a VC or ERF to assess overall impacts are: (i) use a priori weights set by experts and/or decision makers on the relative importance of each indicator, (ii) use empirical weights derived through quantitative analysis of the relationships among indicators (if > 2); or (iii) use the most conservative (i.e., highest calculated impact) state for an indicator as the determinant of the overall impact for the VC or ERF.
2. Degree of dependency among indicators. Indicators are usually treated independently and impacts additively (e.g., McDonald 2000; Gunn and Noble 2009; Canter and Atkinson 2011; Gan et al. 2017), and we followed this additive approach for our main analyses.

Scale is also a critical consideration to avoid in situations where indicators that are signaling localized impacts may become masked by those representing broader scales (Gunn and Noble 2011). In our case study, the Old forest condition VC combines effects of both larger-scaled and smaller-scaled indicators (see Table 1). Therefore, this VC is subject to this masking problem and we addressed it by treating the linear features indicators as independent of Old forest area indicator, and assigned these indicators a weight of 0 until Old forest area was ≤50% of its reference value.

We used an area-weighted approach for aggregating the calculated impacts on indicators in each watershed in order to calculate regional landscape-level summaries of effects at the watershed scale. We calculated area-weighted averages of their estimated values at the raster cell scale for each assessment watershed using Eq. (1):

1 $${\rm{Average}}\,{\rm{impact}}_{VC/ERF} = \frac{{\mathop {\sum}\nolimits_{k = 1}^{nk} {\left( {\left( {\mathop {\sum}\nolimits_{j = 1}^{nj} {{\rm{area}}_j \ast {\rm{impact}}\,{\rm{class}}_{i,j} \ast wt_i} } \right)/\mathop {\sum}\nolimits_{j = 1}^{nj} {{\rm{area}}_j} } \right)} }}{{\# \,{\rm{indicators}}\,{\rm{per}}\,VC\,{\rm{or}}\,ERF_k}}$$

where *n* = the number of watersheds_*j*_ in the assessment area, *k* = the number of indicators_*i*_ in each VC or ERF, and weights (*wt*_*i*_) for each indicator_*i*_ are specified as above.

That is, we weighted the impact values for each indicator by the area of each watershed (“meso-scale”) before averaging the watersheds to report impact at the landscape-level (“macro-scale”) for each VC or ERF. We excluded watersheds in which no projected disturbances occurred on the indicators included in a particular VC or ERF over the time span of the projections. For summaries using indicators directly calculated at the landscape scale, this process simplifies to a non-area-weighted average of impact values for each indicator across all rasters across the landscape.

To examine if and how the impact outcome of aggregation depends on VC compared to ERF, we examined whether the aggregated impact estimated for each watershed was classed the same for each combination of VC compared to ERF, which we termed congruence. We then calculated the % congruence for each VC and ERF combination as the proportion of the study area overlapped by those watersheds classed moderate/high for both the VC and the ERF of a given combination.

We conducted sensitivity analyses to evaluate the effect of uncertainties about how to aggregate impacts for VCs or ERFs: (1) the choice of spatial scale at which indicators are evaluated (watershed vs landscape), (2) the weights applied to each indicator during the aggregation process, and (3) the calculation method of combining impacts from component indicators (e.g., additive vs using the most significant [highest impact]). Results of these tests are reported in Supplementary Information S2.

## Results

### Aggregating Effects into Impacts

The area-weighted mean estimated impact classes for each indicator are shown in Table 2, as are the proportion (%) of study area watersheds classed as minimum (low) or maximum (high) impact. The calculated impacts for all indicators resulted in most of the 142 watersheds that are entirely within the study area being classed low or moderate. The condition indicator “Old forest area” was classed as having a low impact in all watersheds reflecting high levels of old forest protection in the study area. As well, the condition indicator “Hydrological recovery” was also classed as low impact for most watersheds. The condition indicator “Spotted Owl nesting habitat” showed 14% of watersheds classed as in the high impact class. Apparent future loss of Spotted Owl habitat can occur with loss of unprotected younger forest (i.e., forest >110 years of age in drier ecosystems) that is being recruited in modeled future projections as potentially suitable habitat. Furthermore, given that this forest type is outside of protected areas, it is increasingly subject to resource development. Three pressure indicators related to road disturbance (Density of active roads, Density of stream crossings by roads and Road density on coupled steep slopes) and Density of transmission lines classed most watersheds as having either moderate or high impact.

**Table 2:** Summary statistics for the estimated impact classes calculated for each watershed-scale indicator projected to the end year (2112) under the full development scenario

Indicator	Projected indicator value (±SD)^a^	Weighted mean (±SD) impact class of study area	% of study area in minimum (low) impact class	% of study area in maximum (high) impact class
Old forest area (%)	25.9 (±11.1)	1.00 (±0.00)	100.00 (142)	0.00 (0)
Density of active roads (km/km^2^)	0.6 (±0.5)	1.74 (±0.82)	50.80 (86)	24.47 (34)
Density of transmission lines (km/km^2^)	0.1 (±0.2)	1.11 (±0.44)	94.47 (135)	5.17 (6)
Road density (km/km^2^) on coupled steep slopes adjacent to stream)	0.1 (±0.1)	2.04 (±0.95)	42.75 (68)	46.94 (58)
Hydrological recovery (ha/ha)	82.6 (±7.9)	1.05 (±0.27)	97.69 (138)	0.00 (0)
Density of stream crossings by roads (number/km^2^)	0.8 (±0.7)	1.89 (±0.88)	41.91 (66)	35.36 (46)
Spotted Owl nesting habitat area (%)	10.5 (±9.5)	1.39 (±0.72)	74.43 (104)	14.24 (24)

Shown is the mean impact class (±SD) weighted by the area of the watersheds (total *N* = 142) within the boundaries of the study area. In addition, the % of the case study area (and # of watersheds included in the aggregation for each indicator) classed in the minimum (low = 1) and maximum (high = 3) impact classes for each indicator are shown (with the difference being classed as moderate [=2]).

^a^See also Table 3; Sutherland et al. (2016)

Aggregated impacts for VCs or ERFs are presented in Table 3A, B. Table 3A demonstrates that, by considering the aggregation of future projected impacts for watersheds, impacts based on the VC Old forest condition are much higher with shifts of 53.8% of watersheds into the moderate impact class from the low class when based solely on the Old forest area indicator and also accounting for pressure indicators related to road and transmission line development. In contrast, the final impact for the ERF Old forest retention and recruitment did not change because both component indicators (Old forest area and Hydrologic recovery) largely classed watersheds as low impact. We suggest that viewed in terms of VCs, riparian zone areas and conservation of species-at-risk (Spotted Owl) may raise the most concern for managers, followed by the Stream condition and Old forest VCs (Table 3A).

**Table 3:** Estimates of the aggregated impact classes calculated for each valued component and environmental response factor projected to the end year (2112) under the full development scenario

A. Valued components (VCs)					
Valued component (VC)	Indicators	Types	Mean (±SD) impact class	% of study area in minimum impact class	% of study area in maximum impact class
Old forest condition	Old forest area (%) Density of active roads Density of transmission lines	1 C; 2 P	1.28 (±0.54)	46.64 (70)	0.00 (0)
Stream condition	Road density on coupled steep slopes Hydrological recovery	1 C; 1 P	1.53 (±0.78)	40.45 (64)	0.00 (0)
Riparian condition	Density of stream crossings by roads	1 P	1.93 (±0.88)	41.91 (66)	35.36 (46)
Spotted Owl habitat	Spotted Owl nesting habitat area (%)	1 C	1.40 (±0.72)	74.43 (104)	14.24 (24)

B. Environmental response factors (ERFs)					
Environmental response factor (ERF)	Indicator	Types	Weighted mean (±SD) impact class	% of study area in minimum impact class	% of study area in maximum impact class
Old forest retention and recruitment	Old forest area (%) Hydrological recovery	2 C	1.01 (±0.43)	97.69 (142)	0.00 (0)
Road disturbance	Density of active roads Road density on coupled steep slopes Density of stream crossings by roads	3 P	1.56 (±0.13)	41.50 (65)	0.00 (0)
Spotted Owl habitat state	Spotted Owl nesting habitat area (%) Density of transmission lines	1 C, 1 P	1.25 (±0.55)	70.73 (100)	0.81 (1)

Shown are the area-weighted mean impact class (±SD) for each of the watersheds (*N* = 142) wholly contained within the boundaries of the study area. Here we aggregated calculated impacts for each indicator making up the VC (A) or ERF (B) using the equal weighting method among component indicators

Overall for the Road disturbance ERF, >24% of watersheds were assessed as having high impacts related to road development. The Density of transmission line indicator, while linked to the Old forest condition VC is instead included on the Spotted Owl habitat state ERF due to potential negative impacts for this species due to the creation of added edge habitat. Viewed in terms of ERFs, managers may be most concerned with the final projected aggregated impacts due to disturbance from roads and the state of Spotted Owl habitat, although both ERFs were estimated to have only small proportions of the study area classed as high impact (Table 3B).

### Congruence in Interpretations of VC and ERF Combinations

Selection and grouping of the indicators to aggregate, as shown by using the VC compared to ERF examples above, influences the impact estimation from the type and scale of managed area (VC) to the stressors leading to disturbance (ERF) (Table 4). High congruence between the future aggregated impacts projected for some VCs and ERFs occurred, largely owing to these being comprised of similar indicators (Table 4). In particular, watersheds with aggregated impacts projected for the Road disturbance ERF are highly congruent with those projected for three of the four VCs we examined (Old forest condition, Stream condition and Riparian condition), as is the congruence between the Spotted Owl habitat state ERF and the Spotted Owl habitat VC. These results aligned with the interpretations of the individual indicator impacts.

**Table 4:** Congruence between valued components (VCs) and environmental response factors (ERFs) in the area of watersheds projected to be in the aggregated moderate/high impact class by the end year (2112) under the full development scenario

	Valued component (VC)			
Environmental response factor (ERF)	Old forest condition	Stream condition	Riparian condition	Spotted Owl habitat
Old forest retention and recruitment	1.1	3.9	0.0	2.3
Road disturbance	**82.2**	**79.2**	**99.3**	18.6
Spotted Owl habitat state	20.5	25.8	22.1	**87.4**

Shown is the percentage of the area (ha) of the included 142 watersheds in the study area for which each combination of VC and ERF is projected to show an aggregated impact classification of moderate and/or high. Values are: 0.0 = low congruence; 100.0 = complete congruence. See Table 2 caption for methods of selecting watersheds and aggregation of impacts into a mean impact class value for each watershed. Bold indicates high congruence

On the other hand, the Old forest retention and recruitment ERF is only very weakly congruent with any of the selected VCs. This may be due in part to the overall low level of projected impact for this ERF including the Old forest area indicator, which indicated low impact in all watersheds.

In contrast, the amount of Old forest area indicator for the Old forest condition VC included indicators of linear feature disturbance, such as roads and transmission lines within its calculation, while effects of these linear features were kept separate (Road disturbance ERF) and were not included in the Old forest retention and recruitment ERF.

## Discussion

Our study outlines an assessment methodology for making pragmatic, structured analytical decisions to assess cumulative impacts of projected anthropogenic disturbance footprints on ecological values. Strategic modeling of projected disturbance footprints arising from planned resource developments in order to predict and compare their potential cumulative impacts is an approach often used to inform land management decision makers (Recatalá and Sacristán 2014; Mahon and Pelech 2021; Venier et al. 2021). Yet decision makers are challenged to interpret the myriad of potential cumulative effects outcomes for multiple indicators and VCs or ERFs estimated at various spatial and temporal scales. The steps outlined in this methodology are most appropriate for strategic assessments primarily focused on assessing selected ecological effects of projected anthropogenic footprints from forestry and small-scale hydropower developments in forested landscapes. We use temporally modeled scenarios to reflect process uncertainty (i.e., how development activities link to changes in indicators) and to compare the outcomes of different, unknown management futures (Mahon and Pelech 2021). Using our case study we demonstrate, as follows, a number of issues for this type of strategic modeled CEA when selecting appropriate methods to apply.

Scenario-based analyses rely upon explicit quantitative information and assumptions in order to reliably compare the range of future outcomes. Such information is often difficult to obtain or is simply not available, and impacts are instead often evaluated with less quantitative methods (Sizo et al. 2016). Each stage in a CEA process involving inter-linked, complex processes has uncertainties in defining appropriate reference conditions, benchmark values, and aggregation methods (Murray et al. 2018; Hegmann 2019; Singh et al. 2019; Johnson and Ray 2021; Venier et al. 2021). We identified options and trade-offs for customizing spatial decision-support modeling CEAs (see Mahon and Pelech 2021) to address these uncertainties at three key steps: (1) estimating cumulative effects at the indicator level, (2) inferring impact states for each indicator, and (3) aggregating indicators’ impacts by VC or ERF.

One of the most common problems for this type of modeled CEA is the lack of standardized reference values for many of the indicators, in part due to differing degrees of prior developments in areas. Yet the estimation of impacts at all points in the analytical process, whether for individual indicators or combined for a VC or ERF, depends upon the initial choice of reference condition for each indicator (in our case, historical landscape conditions for Old forest area and Spotted Owl habitat state but current conditions for others). This dependency on the reference conditions reinforces the need to carefully specify them a priori and to evaluate their effects on interpretations at different points in the assessment process (Probst and Stelzenmüller 2015).

In calculating impacts, we relied on impact classes and used a combination of broad literature guidance and expert opinion to define class boundaries. Difficulties in determining specific thresholds for the indicators in our case study are consistent with those identified in other studies (Duinker and Greig 2006; Groffman et al. 2006; Johnson 2013; Jones 2016; Hegmann 2019). For example, in forested ecosystems, specifying threshold levels for forest-related indicators (e.g., amounts of habitat and/or late-seral forest, patch sizes of interior forest) are dependent upon the objective of the analysis and the context in which the threshold is defined (Andrén 1994; Dykstra 2004; Price et al. 2007; Venier et al. 2014). Similarly, interdependencies among stream flow, topography, surficial geology, and climate means there is relatively little guidance in the literature on appropriate ecological thresholds for stream and riparian condition indicators (Gergel et al. 2002; Squires and Dubé 2013; Gupta et al. 2021). Expert opinion can be and often is used to guide interpretations of impact in the absence of more indicator-specific information (Opon and Henry 2020). Discrete impact classes using broad and general definitions may not accurately capture true levels of impact, but can provide a practical, comparative approach following a unified logical sequence of decisions.

Aggregation of impacts from multiple indicators to represent impact conclusions at the level of VCs or ERFs can be approached through expert opinion-based aggregation methods, heuristic objective-driven scoring methods, formal statistical methods that seek to maximize weight-of-evidence from multiple indicators (Le Clec’h et al. 2016) or informal statistical methods such as simple additive or other algorithms (e.g., Becker et al. 2017). We used the latter method for our case study example, because it provides a reproducible and generalizable approach amenable to sensitivity testing, particularly given the sparse underlying data on benchmarks and assumptions. However, expert involvement is still required to improve both understanding the implications of aggregated outcomes for the ecological values, as well as to acknowledge both the nature and limitations of the interactions between indicators and the spectrum of risks posed to ecological and social values (Le Clec’h et al. 2016; Johnson and Ray 2021).

By comparing impact results, calculated for both VCs or as ERFs, we were able to explore the effect of uncertainty in how indicators could be grouped together (i.e., methodological uncertainty: Opon and Henry 2020) upon the aggregated overall impact result or conclusion. We confirmed that the importance of impacts signaled by individual indicators could be masked (Duinker and Greig 2006; Therivel and Ross 2007; Bragagnolo and Geneletti 2012) when aggregated to VC unless each indicator is also considered independently or contrasted with alternate groupings, such as the ERFs. Our impact conclusions were generally similar between some VCs and ERFs, which was supported by high spatial congruence between impact statements representing the same ecological value. Yet poor congruence between others occurred because they likely represent different ecological processes, thus interpretations differed about how stressors impact the area.

The extent to which indicators represent either expected deviations from the desired condition or underlying causal processes considered important in changing future ecological conditions (“condition” or “pressure” indicators; Hagan and Whitman 2006) also influences the interpretation of aggregated impacts. Making this distinction about indicator type up-front helps with interpreting the significance of effects when calculating aggregated cumulative impacts, as well as potentially with the choice of appropriate models of indicator interactions (Sutherland et al. 2016). In our case study, aggregated impacts for VCs appeared dominated by those calculated from “condition” indicators, while aggregated impacts of ERFs were dominated by “pressure” indicators. Condition indicators may tend to capture the effect the disturbance footprint has upon the state of the ecological value they represent, while pressure indicators may better alert decision makers to expect changes in ecological functions as particular disturbance activities accumulate. By comparing findings from grouping indicators in multiple ways, considering as we did VCs compared to ERFs, we believe practitioners could deepen their understanding of how impacts arise, and thereby enhance their opportunities to design effective mitigations for undesirable future impacts (see also Coté et al. 2016). Therefore, aggregated impact classifications may usefully signal impact severity and risk but are dependent on indicator grouping, hence choices for aggregation are integral to the assessment process.

Successful application of aggregated impact results in decision making from these landscape modeling types of CEAs requires agreement from decision makers on the parameter values and relative weightings to be used. Such agreement is usually sought through a collaborative process with resource managers or end-users (e.g., Marcot et al. 2012). In turn, this process requires the flexibility embedded in an interative process, as we have proposed here, because decision making at each of the steps may influence the assessment sequence. The proposed approach provides transparency by implementing simple (but considered) assumptions, building on the collective need identified by practitioners for a common framework (e.g., Foley et al. 2017; Mahon and Pelech 2021; Venier et al. 2021) for informing planning decisions, especially when evaluating compliance with policy-defined regulatory targets that are amenable to monitoring as anthropogenic development proceeds.

The approach described here is primarily designed to be used with indicators of anthropogenic disturbance that can be represented as a quantitative value at a particular scale of analysis (e.g., watershed). However, there may be useful indicators that are better represented in other ways, such as maps (Le Clec’h et al. 2016; Hodgson and Halpern 2019). Such indicators could be used in our approach if a method to assess associated impact classes is available, for example using visual scales based on quantitative (e.g., quantiles), qualitative (e.g., expert opinion) criteria (Petter et al. 2013), or showing other types of transformations such as difference maps (for examples of the latter see Supplementary Information S3). Ultimately, however, these indicators may not lend themselves well to aggregation.

### Limitations

In conducting our modeling of cumulative landscape impacts based on anthropogenic disturbance footprints case study, we found that quantitatively combining and aggregating indicator values to allow us to infer potential cumulative impact states at higher levels (i.e., across multiple VCs or ERFs) was significantly challenged in three ways that are directly related to choices of indicators to include:

1. Defining meaningful thresholds or benchmark values for indicators to identify likely significant effects on ecological values. A lack of sufficient data to specify benchmark values for our key indicators (e.g., amount of old forest: Lindenmayer et al. 2005; Price et al. 2007) caused us to rely upon a general definition of impact classes (e.g., low <30%, moderate 30–70%, high >70%) which could potentially misalign with true (but unknown) thresholds, and could mask effects with aggregation.
2. Accounting for the different spatial and temporal scales at which different indicators respond to development activities (e.g., watershed-level versus landscape-level) influences several aspects of the process of aggregating and interpreting impacts across VC and ERFs (Gunn and Noble 2011; Seitz et al. 2011; Venier et al. 2021) in the type of assessment problem we studied. For example, we used the watershed scale as the unit of analysis for evaluating and aggregating impacts on each VC or ERF to apply to the overall landscape, but impacts at other scales (landscape—site) may be just as important in any given CEA. Aggregating into larger spatial units can also lack spatial transparency for individual indicator impacts. Overlaying impact maps for each “indicator × watershed” combination could additionally inform appropriate and effective mitigation designs for individual watersheds.
3. Every step in impact calculation, aggregation and interpretation requires careful thought. Throughout this paper we have noted the effects of each decision step, including choosing indicators to select, choosing groupings of them into VCs or ERFs, specifying relationships between indicators and the potential effects they can have on interpretions of combined impact conclusions about modeled development activities upon ecological values. In a modeling process such as used here, sensitivity analyses (see Supplementary Information S2) can be a useful tool to help practitioners untangle the effects of differing aggregation assumptions upon overall outcomes (Opon and Henry 2020).

Finally, as described above, our case study was limited in the types of anthropogenic disturbance footprints modeled (i.e., primarily focused on forestry and small-scale hydropower developments under natural disturbance conditions). Our selected indicator set and ecological values of interest were focused on those of most decision-making interest in landscapes where resource developments of these types are occurring or are planned to occur. Other aspects of ecosystem function, such as indicators of ecosystem services, are not explicitly accounted for in our VCs or ERFs and this remains an important gap.

## Conclusions

The primary outcome of our research on assessing anthropogenic effects of forestry and related resource developments upon ecological values is to describe a sequence of steps to quantify impacts for a diverse array of indicators. By making considered choices at each step, we demonstrate how to aggregate impacts into VCs or ERFs and how to interpret them. Within these types of assessments, the sequence of steps we propose is iterative; work at a subsequent step could suggest modifications to a previous step, and so on. The scenario-based assessment methodology we present explicitly considers decision trade-offs at each step and enables testing of uncertainties. It does not negate the need for causal-based approaches to validate relationships to improve interpretations over time (e.g., Opon and Henry 2020) consistent with adaptive management principles (Price et al. 2009).

Our methodology for estimating impacts as developed in this study will assist experts and stakeholders through a decision-making process for evaluating outcomes of development activities on VCs or ERFs, given ecological contexts and management objectives. However, the development of operational frameworks for these types of CEAs still require considerable study to improve the quantitative foundations for impact forecasting (McDonald 2000; Coté et al. 2016). The approach we describe is intended to support and encourage this foundational development to better inform planning, monitoring and management.

## Supplementary information

Supplementary Information

## Footnotes

1 Spatially Explicit Landscape Event Simulator (Fall and Fall 2001).

## References

[CR1] Andrén H (1994). Effects of habitat fragmentation on birds and mammals in landscapes with different proportions of suitable habitat: a review. Oikos.

[CR2] Ball M, Somers G, Wilson JE, Tanna R, Chung C, Duro DC, Seitz N (2013). Scale, assessment components, and reference conditions: issues for cumulative effects assessment in Canada. Integ Environ Asses Manag.

[CR3] Beanlands GE, Duinker PN (1984). Lessons from a decade of offshore environmental impact assessment. Ocean Man.

[CR4] Becker W, Saisana M, Paruolo P, Vandecasteele I (2017). Weights and importance in composite indicators: closing the gap. Ecol Ind.

[CR5] Bragagnolo C, Geneletti D (2012). Addressing cumulative effects in strategic environmental assessment of spatial planning. Aestimum.

[CR6] BC Ministry of Forests and Ministry of Environment (1995). Biodiversity Guidebook. Forest Practices Code of British Columbia. https://www.for.gov.bc.ca/hfd/library/documents/bib19715.pdf. Accessed 18 Jan 2022

[CR7] BC Ministry of Forests and Range (2010) Soo TSA timber supply analysis. Public Discussion Paper. BC Mininstry of Forests, Forest Analysis and Inventory Branch, Victoria, B.C. https://www2.gov.bc.ca/assets/gov/farming-natural-resources-and-industry/forestry/stewardship/forest-analysis-inventory/tsr-annual-allowable-cut/soo_tsa_public_discussion.pdf. Accessed 18 Oct 2021

[CR9] Canter LW, Atkinson SF (2011). Multiple uses of indicators and indices in cumulative effects assessment and management. Environ Impact Assess Rev.

[CR8] Canter LW, Ross B (2010). State of practice of cumulative effects assessment and management: the good, the bad and the ugly. Impact Assess Proj Appl.

[CR10] Coté IM, Darling ES, Brown CJ (2016). Interactions among ecosystem stressors and their importance in conservation. Proc R Soc B.

[CR11] Dobbie MJ, Dail D (2013). Robustness and sensitivity of weighting and aggregation in constructing composite indices. Ecol Ind.

[CR12] Dubé MG (2003). Cumulative effect assessment in Canada: a regional framework for aquatic ecosystems. Env Impact Assess Rev.

[CR13] Dubé MG, Duinker P, Greig L (2013). A framework for assessing cumulative effects in watershed: an introduction to Canadian case studies. Integr Environ Assess Manag.

[CR15] Duinker PN, Bubidge EL, Boardley SR, Greig LA (2013) Scientific dimensions of cumulative effects assessment: toward improvements in guidance for practice. Environ Rev 21:40–52. 10.1139/er-2012-0035

[CR14] Duinker PN, Greig LA (2006). The impotence of cumulative effects assessment in Canada: ailments and ideas for redeployment. Environ Manag.

[CR16] Dykstra PR (2004) Thresholds in habitat supply: a review of the literature. BC Min Sustainable Resour Manage, Ecosystem Conserv Section, and BC Min Water Land and Air Protection Biodiversity Branch, Victoria, BC. Wildl Rep No R-27

[CR17] Fall A, Fall J (2001). A domain-specific language for models of landscape dynamics. Ecol Model.

[CR18] Foley MM, Mease LA, Martone RG, Prahler EE, Morrison TH, Murray CC, Wojcik D (2017). The challenges and opportunities in cumulative effects assessment. Environ Impact Assess.

[CR19] Gan X, Fernandez IC, Guo J, Wilson M, Zhao Y, Zhou B, Wu J (2017) When to use what: methods for weighting and aggregating sustainability indicators. Ecol Ind 81:481–492. 10.1016/j.ecolind.2017.05.068

[CR20] Gergel SE, Turner MG, Miller JR, Melack JM, Stanley EH (2002). Landscape indicators of human impacts to riverine systems. Aquat Sci.

[CR21] Groffman PM, Baron JS, Blett T (2006). Ecological thresholds: the key to successful environmental management or an important concept with no practical application?. Ecosystems.

[CR22] Gunn JH, Noble BF (2009). A conceptual basis and methodological framework for regional strategic environmental assessment (R-SEA). Impact Assess Proj Appl.

[CR23] Gunn J, Noble BF (2011). Conceptual and methodological challenges to integrating SEA and cumulative effects assessment. Environ Impact Assess Rev.

[CR24] Gupta A, Farjad B, Wang G, Eum H, Dubé M (2021) Integrated environmental modeling framework for cumulative effects assessment. University of Calgary Press, Calgary, https://prism.ucalgary.ca/bitstream/handle/1880/113082/9781773851990web.pdf

[CR25] Hagan JM, Whitman AA (2006). Biodiversity indicators for sustainable forestry: simplifying complexity. J For.

[CR26] Hegmann G (2019). The insignificance of thresholds in environmental impact assessment: an illustrative case study in Canada: a critique for Environmental Management. Environ Manag.

[CR27] Hodgson EE, Halpern BS (2019). Investigating cumulative effects across ecological scales. Cons Biol.

[CR28] Johnson CJ (2013). Identifying ecological thresholds for regulating human activity: effective conservation or wishful thinking?. Bio Con.

[CR29] Johnson CJ, Ray JC, Blakley JAE, Franks DM (2021). The challenge and opportunity of applying ecological thresholds to environmental assessment decision-making. Handbook of cumulative impact assessment: research handbooks on impact assessment series.

[CR30] Jones FC (2016). Cumulative effects assessment: theoretical underpinnings and big problems. Environ Rev.

[CR31] Joseph C, Zeeg T, Angus D, Usborne A, Mutrie E (2017). Use of significance thresholds to integrate cumulative effects into project-level socio-economic impact assessment in Canada. Environ Impact Assess Rev.

[CR32] Le Clec’h S, Oszwald J, Decaens T, Desjardins T, Dufour S, Grimaldi M, Jegou N, Lavelle P (2016). Mapping multiple ecosystem services indicators: toward an objective-oriented approach. Ecol Ind.

[CR33] Lindenmayer DB, Fischer J, Cunningham RB (2005). Native vegetation cover thresholds associated with species responses. Biol Cons.

[CR34] Mahon CL, Pelech S (2021). Guidance for analytical methods to cumulative effects assessment for terrestrial species. Environ Rev.

[CR35] Marcot BG, Allen CS, Morey S, Shively D, White R (2012). An expert panel approach to assessing potential effects of bull trout reintroduction on federally listed salmonids in the Clackamas River, Oregon. N. A J Fish Manag.

[CR36] McDonald LH (2000). Evaluating and managing cumulative effects: process and constraints. Environ Manag.

[CR37] Murray CC, Wong J, Singh GG (2018). The insignificance of thresholds in environmental impact assessment: an illustrative case study in Canada. Environ Manag.

[CR38] Noble BF, Sheelanere P, Patrick R (2011). Advancing watershed cumulative effects assessment and management: lessons from the South Saskatchewan River watershed, Canada. J Environ Assess Policy Manag.

[CR39] Olagunju AO, Gunn JAE (2015). Selection of valued ecosystem components in cumulative effects assessment: lessons from Canadian road construction projects. Impact Assess Proj Appl.

[CR40] Opon J, Henry M (2020). A multicriteria analytical framework for sustainability evaluation under methodological uncertainties. Environ Impact Assess Rev.

[CR41] Petter M, Mooney S, Maynar SM, Davidson A, Cox M, Horosak I (2013) A methodology to map ecosystem functions to support ecosystem services assessments. Ecol Soc 18(1):31. 10.5751/ES-05260-180131

[CR42] Price K, Daust D (2009) Making monitoring manageable: a framework to guide learning. Can J Res 39:1881–1892. 10.1139/X09-101

[CR60] Price K, Holt R, Kremsater LL (2007) Representative forest targets: informing threshold refinement with science. Unpublished review paper prepared for JSP and CFCI. 7 June 2007. https://www2.gov.bc.ca/assets/gov/farming-natural-resources-and-industry/natural-resource-use/land-water-use/crown-land/land-use-plans-and-objectives/westcoast-region/great-bear-rainforest/ei01_final_report.pdf

[CR43] Price K, Roburn A, MacKinnon A (2009). Ecosystem-based management in the Great Bear Rainforest. Ecol Mgmt.

[CR44] Probst WM, Stelzenmüller V (2015). A benchmarking and assessment framework to operationalize ecological indicators based on time series analysis. Ecol Ind.

[CR45] Recatalá L, Sacristán D (2014). A minimum indicator set for assessing resources quality and environmental impacts at planning level in a representative area of the European Mediterranean Region. Ecol Ind.

[CR46] Reynoldson TB, Norris RH, Resh VH, Day KE, Rosenburg DM (1997) The reference condition: a comparison of multimetric and multivariate approaches to assess water-quality impairment using benthic macroinvertebrates. J N. A Benth Soc 16:833–852

[CR47] Seiler A, Folkeson L (eds) (2006) Habitat fragmentation due to transportation infrastructure – COST 341 national state-of-the-art report Sweden. http://www.vti.se/publications. Accessed 9 Mar 2022

[CR48] Seitz NE, Westbrook CJ, Noble BF (2011) Bringing science into river systems cumulative effects assessment practice. Env Imp Ass Rev 31:172–179. 10.1016/j.eiar.2010.08.001

[CR49] Sinclair AJ, Doelle M, Duinker PN (2017). Looking up, down, and sideways: reconceiving cumulative effects assessment as a mindset. Environ Impact Assess Rev.

[CR50] Singh GG, Lerner J, Murray CC (2019). Response to critique of “The insignificance of thresholds in environmental impact assessment: an illustrative case study in Canada”. Environ Manag.

[CR51] Sizo A, Noble BF, Bell S (2016). Strategic environmental sssessment framework for landscape-based, temporal analysis of wetland change in urban environments. Environ Manag.

[CR52] Squires AJ, Dubé MG (2013). Development of an effects-based approach for watershed scale aquatic cumulative effects assessment. Integr Environ Assess Manag.

[CR53] Stoddard JL, Larsen DP, Hawkins CP, Johnson RK, Norris RH (2006). Setting expectations for the ecological condition of streams: the concept of reference condition. Ecol Appl.

[CR54] Sutherland GD, O’Brien DT, Fall SA, Waterhouse FL, Harestad AS, Buchanan JB (eds) (2007) A framework to support landscape analyses of habitat supply and effects on populations of forest-dwelling species: a case study based on the Northern Spotted Owl. BC Min Forests and Range, Research Branch, Victoria, BC, Tech Rep 038. http://www.for.gov.bc.ca/hfd/pubs/Docs/Tr/Tr038.htm

[CR55] Sutherland GD, Waterhouse FL, Smith J, Saunders SC, Paige K, Malt J (2016). Developing a systematic simulation-based approach for selecting indicators in strategic cumulative effects assessments with multiple environmental valued components. Ecol Ind.

[CR56] Therivel R, Ross B (2007). Cumulative effects assessment: does scale matter?. Env Imp Assess Rev.

[CR57] Venier LA, Thompson ID, Fleming R (2014). Effects of natural resource development on the terrestrial biodiversity of Canadian boreal forests. Environ Rev.

[CR58] Venier LA, Walton R, Brandt JP (2021). Scientific considerations and challenges for addressing cumulative effects in forest landscapes in Canada. Environ Rev.

[CR59] Wu K-K, Zhang L-P, Fang Q-H (2015) An approach and methodology of environmental risk assessment for strategic decision-making. J Env Assess Pol Manage 16. 10.1142/S1464333214500136

