# Supplementary material for: A Pragmatic Approach for Developing Landbase Cumulative Effects Assessments with Aggregated Impacts Crossing Multiple Ecological Values
Source: Environ Manage. 2022 Mar 28;69(5):1020–34. doi: 10.1007/s00267-022-01632-9 (PMC9038830; doi:10.1007/s00267-022-01632-9)
Supplement: Supplementary file 1 — Supplementary Information [file 267_2022_1632_MOESM1_ESM.docx]

**Sutherland GD, Smith J., Waterhouse FL, Saunders SC, Paige K**. A pragmatic method for aggregating and interpreting projected impacts crossing multiple scales and values in strategic cumulative effects assessments. Environmental Management. Corresponding author: Glenn D. Sutherland, Wildlife Infometrics Inc. email: [glenn.sutherland@wildlifeinfometrics.com](http://glenn.sutherland@wildlifeinfometrics.com)

# S-1. Spatio-Temporal Projection Models of Landscape Dynamics

In this CEA case study we applied, as follows, six previously developed raster-based spatio-temporal models to simulate changes in landscapes, including forest growth, anthropogenic disturbances, and generation of indicators (Sutherland et al. 2007, Sutherland et al. 2016 Supplemental Materials: Appendix A).

## Forest Growth Projection

The dominant vegetation type of the study area is forest stands mapped and classified by dominant (leading) and co-dominant (secondary) species of trees in the source database (VRI: Vegetation Resource Inventory^[[1]](#footnote-1)^). Stands may cover multiple raster cells/stand. For each stand, we projected two attributes annually: tree age and tree height. Age is incremented annually unless a disturbance resets the age. Tree heights are projected using a Variable Density Yield Projection tool (VDYP 6^[[2]](#footnote-2)^) which incorporates information on a site’s potential for tree growth (site index), the dominant and co-dominant tree species growing on the site, and the forest inventory ecological classification (Table S-1). For the purposes of height calculation, the tree species composition on a site is assumed to be unaffected by natural disturbances and forest harvesting. Note that detailed vegetation succession modeling is not undertaken in this model.

## Forestry Harvest Block Projection

We did not implement a fully spatial harvesting model, but instead used an externally generated spatial schedule of harvested blocks produced by the B.C. Ministry of Forests Lands and Natural Resource Operations for the study area (BC Ministry of Forests and Range 2010). This approach allowed our assumptions to be consistent with the current (2012) forest management assumptions for the study area. Based on the harvest schedule, stand height and age of blocks scheduled to be cut each year were preset to the appropriate age and height assumed by the management prescription for that area. Note that this model is a deterministic (i.e. a non-stochastic) implementation of forest harvesting dynamics.

Two limitations of using an external harvest schedule were: (1) only one spatial outcome (i.e. proposed pattern of cutblocks) is used in the scenario analysis; and (2) dynamic interactions between forest harvesting and other stochastic and/or deterministic disturbances are not fully captured.

## Projection of Natural Disturbances

We used a stochastic model which projects stand-replacing disturbances, for example, moderate to severe wildfires. The mean area disturbed per disturbance event each year and the frequency of events per year (Table S-1) were estimated based on landscape scale parameters describing the expected range of natural variation in seral stage distributions (British Columbia Ministry of Forests and British Columbia Ministry of Environment 1995).

Simulation of a stand-replacing natural disturbance event proceeds as follows. First a specified number of fires are initiated in random raster cells within each biogeoclimatic zone based on the area of the variant and the fire return frequency of the zone (Table S-1). The latter is drawn from a normal distribution of fire return intervals with the means varying by BEC variant and the SD specified as 40% of the mean (Table S-1). Fires are spread from initiation points until a maximum size is reached (Table S-1). Additional stochastic behaviour determining fire shape is implemented to reflect the local variability in fire behaviour due to local conditions during a fire event (Sutherland et al. 2007). Fire control or suppression effects were scenario specific.

Table S-1. Key natural disturbance parameters by BEC zone showing estimates derived from British Columbia Ministry of Forests and British Columbia Ministry of Environment 1995^1^ and also Sutherland et al. (2007).

| BEC zone^2^ | Estimated frequency of stand-replacing events (mean annual rate = 1/frequency) | | Applied mean disturbance patch size (ranges among BEC variants) |
| --- | --- | --- | --- |
|  | Expert opinion or  summary data | Empirical |  |
| BG | 200 | 700 | 27.5 |
| CWH | 1000 | - | 0.2–510 |
| ESSF | 2000 | 565 | 12–517 |
| IDF | 300 | 545 | 11–275 |
| MH | 2000 | 935 | 7–100 |
| MS | 1000 | 940 | 12–517 |
| PP | 200 | 1820 | 17 |

^1^ the Biodiversity Guidebook (B.C. Ministry of Forests and B.C. Ministry of Environment 1995).

^2^ Biogeoclimatic Zone classifications used in BC are described in Meidinger and Pojar (1991).

## Run-of-River (RoR) Facilities Projection

This RoR projection model is intended as a tool for exploring the relative impact of different levels of RoR development rather than a predictor of the actual future development trajectory. We projected annually the spatial dispersion of run-of-the-river (RoR) power generation sites (locations and footprints) using estimated demand, proximity to existing transmission line infrastructure and bio-physical characteristics of the site and the surrounding watershed.

First potential ROR locations are identified, based on active water license applications related to power generation for a given year. Then a 20% annual relative probability of a RoR being established is applied to select locations from the map each year by assuming an equal relative probability for all potential (yet unbuilt) sites in the first 20 years. After 20 years, sites are selected using a relative probability of development based on cost per kilowatt (including transmission line infrastructure) for connecting the development to the 2012 transmission grid, as predicted by a previously compiled assessment of sites with run-of-river hydro potential (BC Hydro 2011). The average lifespan for an ROR is assumed to be 35 years, after which it is decommissioned. Once a RoR site is selected, development is broken into a series of phases and impacts can be specified for each.

Note that this is a simplified model for projecting RoR installations and their operations and does not account for additional factors such as multiple installations on connected stream networks.

## Projection of Access Features Connecting Infrastructures

Access to infrastructures is projected as the minimum set of infrastructure (roads and transmission lines) required to connect all developments produced over the full modeling period to the existing infrastructure by using graph theory (Fall et al. 2007). First, a base input layer for each infrastructure type is created consisting of existing access corridors and of developments requiring access of that type. A minimum planar graph (MPG) is then extracted from this patch layer using least cost path links, such that all patches are linked to all other patches, no links cross, and the set of links are the minimum cost to link all patches together (Fall et al. 2007). Finally, the subset of the MPG is extracted that represents the minimum set of links (e.g., roads) required to link all developments to existing access infrastructure. We used this subset of new access corridors to update the infrastructure network. Roads are projected before transmission lines, because transmission line siting is typically influenced by the prior presence of roads.

Calculation of the least cost path uses a relative cost surface parameterized to represent biophysical or policy constraints on development. In this case study, higher cost values are assigned to lakes, rivers, streams, parks, and steep slopes (> 45 degrees). Lower cost values are assigned to existing and new linear developments and to slopes < 5 degrees. Intermediate cost values are assigned to intermediate slopes and wildlife or ecological features with policy restrictions on adjacency to access. In addition, we assumed future linear developments will follow, where possible previous developments. See Sutherland et al. (2007) for further description of how cost surfaces are modelled.

## Indicator Generation Model

We generated a variety of indicator types for the study. Presented results in the main paper are areal. Areal indicators measure areas of a feature, or lengths for linear features (e.g., roads). Scaling factors are applied to estimate linear features in our raster-based model analysis framework to account for differences in raster width relative to the feature and the imposed stepped geometry of the feature.

Indicators are tracked for any combination of strata (e.g., management units such as watersheds or the operable forest area, wildlife habitat management areas, slope classes, or zones by elevation) in the analysis framework. The steps involved in indicator generation occur after the landscape projection models are executed for the specified number of replicates and time periods (see main paper). The response indicators defined for the analysis are then calculated using the sequence of model steps, as follows:

1. For each time step, the model estimates the densities of stream crossings for each of the strata defined for the analysis (e.g., watershed, slope class, biogeoclimatic zone, forest management zone).
2. For each time step, the model estimates the indicator values for each of the strata at the raster cell scale, followed by a summary estimate of indicator values at the watershed and landscape scales.
3. For each time step, the model calculates the mapped indicators involving streams (e.g., RoR developments) at the watershed scale, and their summary at the landscape scale.

Finally, the indicator model generates the tables (and in some cases maps) of the response indicator values by strata and time-period that are used as input into the post-processing analyses described in the main paper (see Fig. 1).

## References Cited

BC Hydro (2011) <https://www.bchydro.com/toolbar/about/planning-for-our-future/irp/current-plan/document-centre/reports/final-ror.html>. Accessed January 28, 2022

BC Ministry of Forests and Ministry of Environment (1995) Biodiversity Guidebook. Forest Practices Code of British Columbia. https://www.for.gov.bc.ca/hfd/library/documents/bib19715.pdf

Meidinger D, Pojar J (eds) (1991) (eds) Ecosystems of British Columbia. British Columbia Min of Forests, Research Branch, Victoria, BC Special Rep Series 6. <https://www.for.gov.bc.ca/hfd/pubs/Docs/Srs/Srs06.pdf>

Sutherland GD, O`Brien DT, Fall SA, Waterhouse FL, Harestad AS, Buchanan JB (eds) (2007) A framework to support landscape analyses of habitat supply and effects on populations of forest-dwelling species: a case study based on the Northern Spotted Owl. BC Min Forests and Range, Research Branch, Victoria, BC Tech Rep 038. [http://www.for.gov.bc.ca/hfd/pubs/Docs/Tr/Tr038.htm](http://www.for.gov.bc.ca/hfd/pubs/Docs/Tr/Tr038.htm.%20Accessed%2018%20Nov%202014)

# S-2. Description of Scenarios and Sensitivity Analyses

## Scenario Specification

Two scenarios were used for analyses (Table S2-1):

Table S2-1. Scenarios defined for the analyses presented in the main paper were used in sensitivity analyses below.

| **Purpose** | **Scenario Name** | **Scenario Components** | **Description** |
| --- | --- | --- | --- |
| Reference value | LTE^1^ | Long-term equilibrium (no development); natural disturbance (ND) with no fire suppression | Historical landscape generated for a reference conditions in the modelled study area^2^ |
| Impact comparisons | Full Development | Harvesting^1^; RoR+ ND with fire suppression | 2012-2112 projection of landscape to calculate cumulative impact case study |

^1^ BC Ministry of Forests and Range 2010

^2^ Simulations were run for 1100 yrs. The final 100 years were used to represent ‘equilibrium’ conditions and used as the reference condition.

In this case study, our use of a pre-determined harvest schedule prevented estimation of variances among scenarios. The analyses we present are therefore based upon randomly seeded single iterations of each scenario.

## Sensitivity Analyses of Key Aggregation Assumptions

Aggregation methods in impact assessments are subject to several uncertainties, including the choice of spatial scale at which indicators are evaluated, the weights applied to each indicator during the aggregation process, and the calculation method of combining impacts, all reflecting types of uncertainty in how the indicators may be functionally related (Gan et al. 2017; Opon and Henry 2020).

Below, we present results of sensitivity tests designed to explore some implications of these assumptions on aggregated impact conclusions used for the case study area. We present the mapped results, if possible, to demonstrate changes in calculated impact classes for each watershed in the study area.

### Assumption #1 – Benchmark Values for Each Indicator and Effects of Spatial Scale

Our default criteria for calculating aggregated impacts (1=Low; 3=High) for the Old Forest Condition VC under the full development scenario (Table S2-1) are as follows:

1. impact states for each watershed are averaged over all included indicators; “pressure” indicators are only included in the aggregated impact estimate if amount of old forest is less than a criterion value (in this study until the ‘Old forest area’ indicator was $\leq$50% of its reference value);
2. equal weighting is given to the value of each indicator in the aggregation; and
3. benchmark values are calculated from the LTE landscape for “condition” indicators and are calculated using the current landscape for “pressure” indicators.

We examine scale sensitivities by presenting results for three time periods, as follows:

- 1. ‘watershed average; pressure-landscape average’ (default) reference values for the “condition” indicators are compared to the average value for each watershed for the reference landscape whereas “pressure” indicators to the landscape average (Fig. S2-1
  2. ‘all indicators-landscape average’ reference values for both the “condition” and “pressure” indicators are compared to the reference landscape average value as a common spatial scale for those indicators (Fig. S2-2)
  3. ‘all indicators-watershed average’ reference values for both the “condition” and “pressure” indicators are compared against the watershed average value on the reference landscape (Fig. S2-3).

We found that choice of scale for calculating the reference value had a marked effect on the distribution and level of impacts among watersheds at the VC level (Fig. S2-1 compared to Figs. S2-2 and S2-3). Using the ‘all indicators-landscape average’ (Fig. S2-2), the overall level of impacts was broadly similar between this criterion and the default (Fig. S2-1), but the spatial distribution of impact states differed. Watersheds near the main river systems were frequently classed as having a higher level of impact under the default criteria— ‘watershed average; pressure-landscape average’— than under the ‘all indicators-landscape average’ criteria; whereas other watersheds showed the opposite (compare Fig. S2-2 with Fig. S2-1). In contrast, the ‘all indicators -watershed average’ criteria produced both a lower level of total impact, and a strongly different spatial patterning of impacts (compare Fig. S2-3 with Fig. S2-1).

### Assumption #2 –Effects of Weightings on Each Indicator on Aggregated Impact States

We examined the effect of altering the weight each indicator has on the aggregated (rolled-up) impact state for the VC by applying the weights shown in Table S2-1 for the LTE vs full development scenario. Alternate weights were chosen to emphasize amounts of old forest relative to other indicators in the aggregation calculations.

Table S2-1. Weights applied to each indicator used to aggregate to the Old Forest Condition VC in sensitivity testing.

| Indicator | Default Weight | Alternate Weight |
| --- | --- | --- |
| Area of old forest | 1.0 | 1.0 |
| Area of interior habitat | 1.0 | 0.8 |
| Road density | 1.0 | 0.7 |
| Transmission line density | 1.0 | 0.5 |

We found that re-weighting the individual indicators has a much smaller effect on the estimated aggregated value than did the choice of which scale to use (landscape vs watershed) as the reference value. Differences between average aggregated impacts ranged between 1-1.5% likely due to the dominant effect old forest has at the VC level.


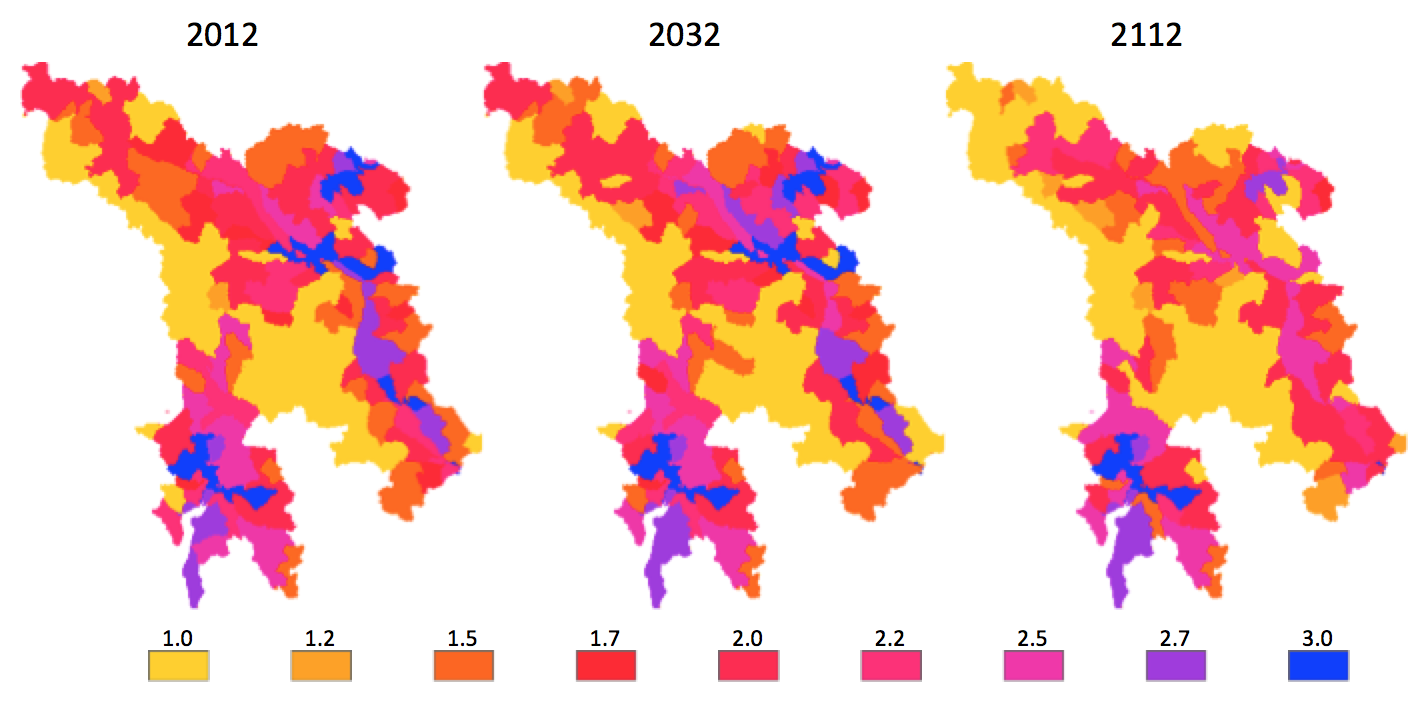


Fig. S2-1. Maps of the combined (rolled-up) impact states for the Old Forest Condition VC under the full development scenario using default decision criteria for aggregating indicator impacts (i.e., ‘watershed average; pressure-landscape average’ scenario).

.


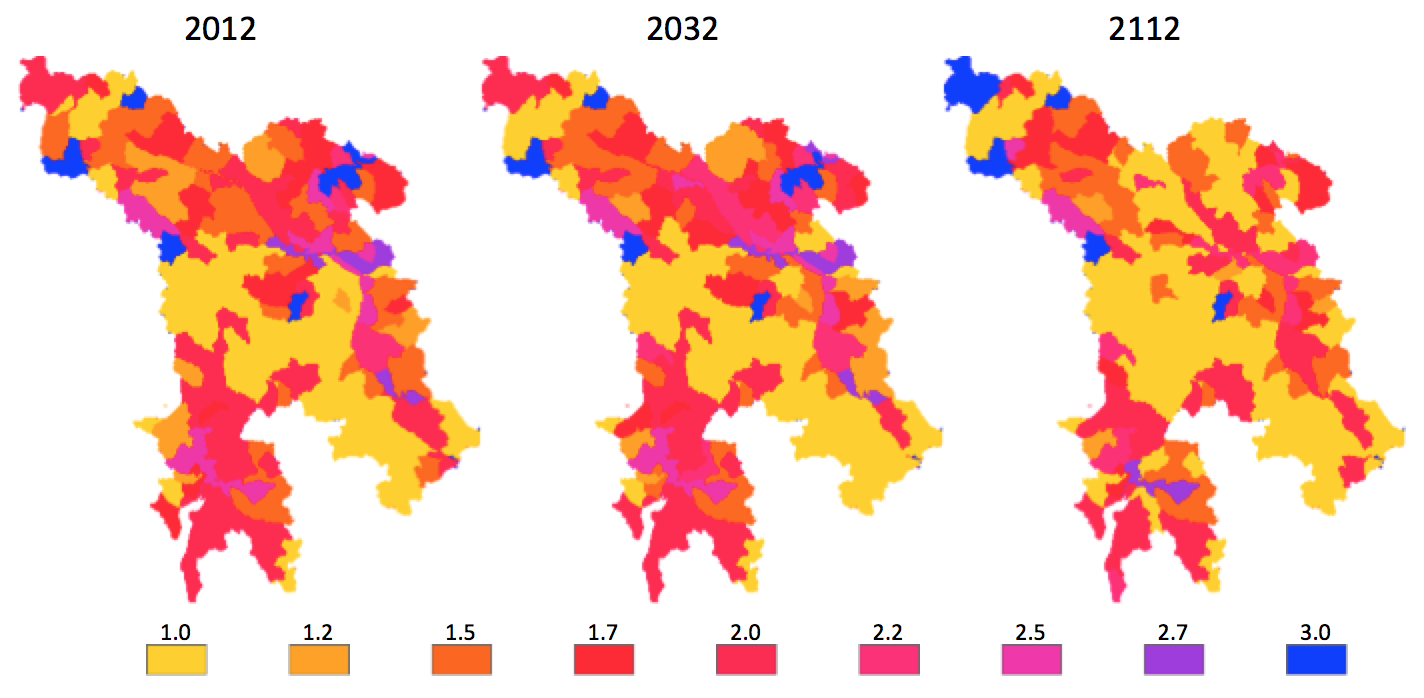

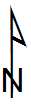


Fig. S2-2. Maps of the aggregated (rolled-up) impact states for the Old Forest Condition VC under the full development scenario using a common scale (landscape average) for benchmark values among-indicators used for aggregating indicator impacts. (i.e., ‘all indicators-landscape average’).

The criteria used to create these maps are the same as Fig. S2-1, except that that both the “condition” and “pressure” indicators were benchmarked against the landscape average value on the reference landscape for those indicators.


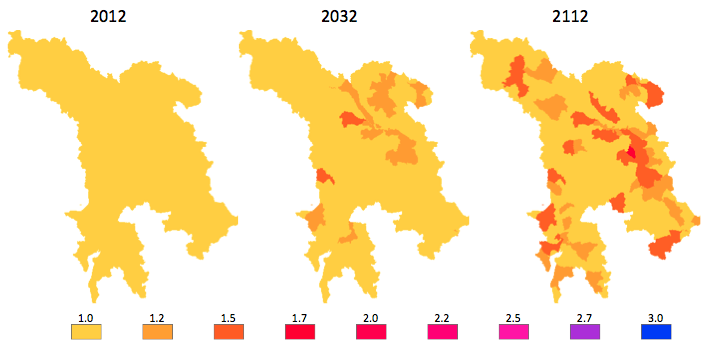

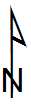


Fig. S2-3. Maps of the combined (rolled-up) impact states for the Old Forest Condition VC under the full development scenario using a common scale (watershed average) for benchmark values among-indicators used for aggregating indicator impacts (i.e., ‘all indicators -watershed average’).

The criteria used to create these maps are the same as Fig. S2-1, except that that both the “condition” and “pressure” indicators were benchmarked against the watershed average value on the reference landscape for those indicators.

### Assumption #3 –Effects of Calculation Method on Aggregated Impact States

Finally, we examined the sensitivity of aggregated impacts on VCs or ERFs to the method of aggregating individual impacts. Our default assumption was to combine impacts in an additive way (e.g., as an average of the indicator-specific impacts). An alternative assumption is to consider the highest impact at the indicator level as the most significant determinant of all impacts at the VC level.

Accordingly, we rolled-up impacts expected under the full development scenario (Table S2-1) for the Old Forest Condition VC choosing the most significant (conservative) impact of the four indicators as the aggregated value and found VC impact levels were increased from 6.7% to 66% using the maximum impact over the average impact. The effects of using the maximum interacted strongly with the scale at which benchmark values were calculated. For example, the case in which all indicator impact states are calculated at the watershed level showed the largest increase in the VC impact state (66%), and the opposite case in which all indicator impact states are calculated at the landscape scale showed the least change in impact (6.7%) at the VC level.

## References Cited

British Columbia Ministry of Forests and Range (2010) Soo TSA timber supply analysis. public discussion paper. BC Min of Forests, Forest Analysis and Inventory Branch, Victoria, B.C. [https://www2.gov.bc.ca/assets/gov/farming-natural-resources-and-industry/forestry/stewardship/forest-analysis-inventory/tsr-annual-allowable-cut/soo_tsa_public_discussion.pdf. Accessed 21 Jan 2022](https://www2.gov.bc.ca/assets/gov/farming-natural-resources-and-industry/forestry/stewardship/forest-analysis-inventory/tsr-annual-allowable-cut/soo_tsa_public_discussion.pdf.%20Accessed%2021%20Jan%202022)

Gan X, Fernandez IC, Guo J, Wilson M, Zhao Y, Zhou B, Wu J. (2017) When to use what: methods for weighting and aggregating sustainability indicators. Ecol Ind 81:481-492. https://doi.org/10.1016/j.ecolind.2017.05.068

Opon J, Henry M. (2020) A multicriteria analytical framework for sustainability evaluation under methodological uncertainties. Environ Impact Assess Rev 83.106403, <https://doi.org/10.1016/j.eiar.2020.106403>

# S3. Mapping Changes in Calculated Impacts

## Example of non-forest alternate indicators for strategic modeling

Comparing differences in mapped values or states for any of the output indicators or VC/ERFs is an informative way to assess where the relative impacts are changing most significantly on the landscape through time. We illustrate this concept with a difference map of projected changes in a single “pressure” indicator (Fig. S3-1 hydrological recovery) calculated under the full development scenario relative to the reference condition for this indicator. With this type of map, it is possible to track geographic shifts in values of indicators because of development patterns.


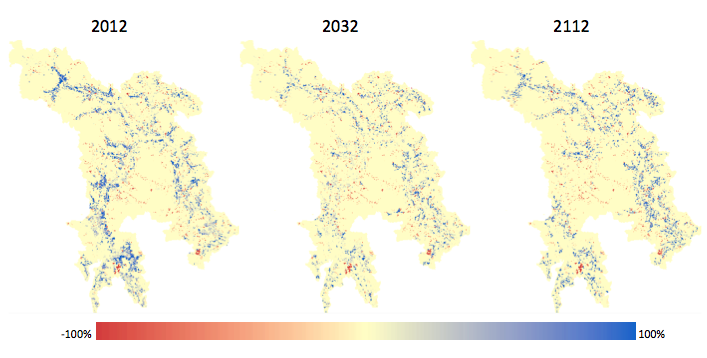

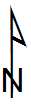


Fig. S3-1. Illustrative example of a set of difference maps for the hydrological recovery indicator.

Shown are difference between the value at each cell (pixel) between the projected indicator value at three different time periods under the full development scenario and the benchmark value. Deviations in this case are calculated from the historical reference landscape condition (LTE). Red indicates a negative effect hence increasing impacts relative to the benchmark value, while blue indicated a positive value ie., low or no impact relative to the benchmark value. Note that other benchmark values (e.g., calculated from the current landscape condition) could also be used.

1. Available at <https://pub.data.gov.bc.ca/datasets/02dba161-fdb7-48ae-a4bb-bd6ef017c36d/2010> [online: accessed Jan. 21, 2022] [↑](#footnote-ref-1)
2. Current versions of the VDYP stand projection software are available at: <https://www2.gov.bc.ca/gov/content/industry/forestry/managing-our-forest-resources/forest-inventory/field-forms-and-software/software-download> [online: accessed Jan. 21, 2022] [↑](#footnote-ref-2)
